# The Coalition Against Typhoid: Mobilizing a Community for a Global Fight

**DOI:** 10.1093/cid/ciy1117

**Published:** 2019-03-07

**Authors:** Sarah Lindsay, Bruce Gellin, Alice Lee, Denise Garrett

**Affiliations:** Sabin Vaccine Institute, Washington, District of Columbia

**Keywords:** typhoid, advocacy, social mobilization, prevention and control

## Abstract

Typhoid became a low priority on the global public health agenda when it was largely eliminated from developed countries in the 1940s. However, communities in South Asia and sub-Saharan Africa continue to bear the brunt of the disease burden. One strategy to increase attention and coordinate action is the creation of a coalition to act as a steward for typhoid. The Coalition against Typhoid (CaT) was created in 2010 with the mission of preventing typhoid among vulnerable populations through research, education, and advocacy. CaT successfully raised the profile of typhoid through convening the community with a biennial international conference that has experienced growing participation, disseminating data and news through a website and newsletter with increasing readership, and advocating through social media and a blog reaching a diverse audience. In 2017, CaT joined forces with the Typhoid Vaccine Acceleration Consortium to “Take on Typhoid,” combining advocacy and communications efforts to mobilize researchers, clinicians, and decision makers at the global, regional, and local levels to introduce the new typhoid conjugate vaccine. As a result, the knowledge base, political will, and momentum are increasingly in place to implement prevention and control interventions including the typhoid conjugate vaccine in the poor communities that have historically been left behind.

Typhoid is not a disease of the past; it is a disease of the poor. In communities with access to clean water and improved sanitation, typhoid is rare. But in the many areas of the world where these are dreams, not even luxuries, the threat of typhoid persists. Coupled with the growing threat of antibiotic-resistant strains, this disease of the poor is becoming increasingly difficult to treat. There is an urgent public health need to expand preventive measures that can slow the development of drug resistance and reduce the impact of typhoid.

Despite resulting in 12 million cases and >128 000 deaths globally each year, typhoid, a bacterial infection caused by *Salmonella enterica* subspecies *enterica* serovar Typhi, has remained low on the global public health agenda since the 1940s when the disease became a rarity in developed countries [1, 2]. As noted in *The Lancet* in 2012, “whoever invented the phrase ‘out of sight, out of mind’ must have been thinking of typhoid fever” [3]. Efforts to prevent and control typhoid emerged more than a century ago, yet attention and prioritization of the disease have since diminished, leaving poor communities in South Asia and sub-Saharan Africa to bear the brunt of the morbidity and mortality of typhoid [3].

## Launching the Coalition Against Typhoid

Combating a disease that keeps such a low profile presents an inherent challenge. One global strategy that has been employed to redirect funds and research efforts to the overlooked health problems facing low- and middle-income countries like typhoid is the creation of coalitions [4]. Coalitions act as conveners of a specific health issue by providing a forum to the many sectors that have a stake in the issue to take a stand together by advocating for funding, mobilizing resources for research, sharing knowledge, and fostering capacity development. With a shared vision, coalitions can improve effectiveness and efficiency by strengthening partnerships and consolidating valuable resources to collaborate on research and implementation issues, share success stories, and convey viewpoints to global health and development decision makers [4].

To raise the profile of typhoid on the global health agenda, the Coalition against Typhoid (CaT), with support from the Bill & Melinda Gates Foundation, was created in 2010 with the mission of preventing typhoid among vulnerable populations through research, education, and advocacy. CaT acts as a catalyst in the global health and development community and supports prevention and control interventions by coordinating partnerships, convening decision makers, and advocating for sustainable solutions to typhoid, including access to clean water and vaccines for vulnerable populations.

## Coalition Structure

CaT is made up of individual members—people who share the common goal to prevent typhoid. Over the course of 8 years of work, CaT recruited >1000 members across different sectors including health; engineering; water, sanitation, and hygiene; and human rights. Prior to 2016, CaT consisted of organizational members. However, with multiple disease priorities fighting for one organization’s attention and with staff turnover at organizations, this model was not effective. By broadening membership to individuals, CaT was able to garner multiple champions from within one organization, as well as individual members from organizations without a specific mandate for typhoid control. This broad membership base enables CaT to raise awareness among members of the global health and development community not previously active in typhoid prevention.

An important factor in CaT’s success is that the membership reflects the population that typhoid impacts most. While CaT members come from >75 countries all over the world, the majority of members are based in regions that bear the disease burden of typhoid. Thirty-five percent of CaT members are based in South or Southeast Asia and 23% are based in Africa. CaT ensures it is responsive to this diverse membership through a multistakeholder steering committee. Additionally, CaT receives member feedback through the CaT website and in-person side events, and by making outreach calls to members to tailor activities to benefit its membership.

## Coalition Impact

CaT has successfully raised the profile of typhoid and is continuing to advance prevention and control efforts. Both in person and digitally, CaT has engaged and mobilized researchers, scientists, and advocates to coordinate efforts in the fight against typhoid and deliver results. CaT has convened the community through a biennial CaT conference with growing participation, disseminated data and news through a website and newsletter with increasing readership, and advocated through social media and a blog reaching a diverse audience [5]. CaT’s work has grown even more important throughout the last year when it joined forces with the Typhoid Vaccine Acceleration Consortium (TyVAC). Together CaT and TyVAC implemented the “Take on Typhoid” campaign to disseminate the latest evidence to global and national decision makers. Take on Typhoid has focused attention on typhoid as the new typhoid conjugate vaccine (TCV) was prequalified and recommended by the World Health Organization (WHO) and Gavi, the Vaccine Alliance opened a funding window for eligible countries to receive support for TCV introduction. After 8 years of work, the conversation around typhoid is no longer *whether* and *how* to stop it, but rather *when* and *where* [6].

## Convening the Community

Every 2 years, CaT hosts the only international conference on typhoid and other invasive salmonelloses. Participants attend with the aim of disseminating new data, sharing and promoting best practices for prevention and control, fostering partnerships across sectors and regions, and equipping colleagues with tools to contribute to national, regional, and global advocacy efforts. The 10th and most recent International Conference on Typhoid and Other Invasive Salmonelloses took place in Kampala, Uganda, in April 2017 and featured conversations on topics including disease burden, antimicrobial resistance, updates on the vaccine pipeline from manufacturers, and discussions of global policy from public sector representatives [7]. The conference was attended by a diverse audience of >250 participants from 38 countries, primarily from Africa and Asia. Along with plenary and symposia sessions, the scientific agenda included 95 original poster abstracts, a 120% increase from the Ninth International Conference on Typhoid and Other Invasive Salmonelloses.

The research, evidence, and ideas shared in Kampala helped provide a foundation for global action against typhoid. With a new TCV developed in 2017, the time was ripe for renewed global interest in updating policies for typhoid prevention and control. In October 2017 the WHO’s Strategic Advisory Group of Experts Typhoid Working Group reviewed select evidence presented at the conference, including data sets from surveillance projects like the Surveillance of Enteric Fever in Asia Project, in updating recommendations for typhoid vaccination [8]. Additionally, results from the conference survey, administered at the conference and receiving a 66% response rate, illustrated the success of the conference. The majority of participants reported leaving with renewed excitement and energy toward the goal of reducing the global typhoid burden. They also went home with new ideas. Eighty-eight percent of respondents said they would implement techniques learned at this conference in their practice or work, and 86% agreed that they learned something from another region that could be applied in their own. The next International Conference on Typhoid and Other Invasive Salmonelloses will take place in March 2019 in Vietnam. However, between the international conferences, the energy and enthusiasm of the typhoid community need regular encouragement to sustain member mobilization. CaT continues to build momentum by regularly bringing together CaT members at global venues such as the American Society for Tropical Medicine and Hygiene annual meeting and similar fora.

## Website and Newsletter

Disseminating data and facilitating knowledge sharing are important avenues to keep a community informed and to reach decision makers with actionable evidence. Since CaT joined forces with TyVAC to maximize advocacy and communications efforts, the partnership has harnessed multiple communications channels to help bring attention to typhoid and raise awareness of prevention and control efforts. The Take on Typhoid website consolidates several resources for use by advocates when meeting with decision makers, including news on outbreaks and the increasing danger of antimicrobial resistance, information on the TCV, and the latest research. The website was also an important tool to disseminate groundbreaking policy decisions. When the first TCV (Typbar-TCV) achieved WHO prequalification and the WHO recommended its use, CaT issued press releases on the website. The press releases facilitated the placement of stories about these policy decisions in >40 news outlets [9].

The website also serves as a continuing resource for advocates, researchers, and policy makers, with fact sheets, infographics, and message maps, many of which are tailored by country or region. While national decisions around vaccine introduction are encompassed within numerous complicated socioeconomic and political realities, translating data around disease burden and cost-effectiveness of vaccine introduction can help inform policy decisions. The website is also regarded as a starting point for new researchers entering the field of typhoid, paratyphoid, or nontyphoidal *Salmonella* research for the first time. Of the 14 856 unique website visitors during September 2017–July 2018, 89% were new to the site. The Take on Typhoid knowledge exchange newsletter, meanwhile, informs a broader audience on a bimonthly basis of research, outbreaks, news stories, and vaccine updates. CaT members actively contribute to the newsletter by submitting articles to be featured. The newsletter is another integral tool for disseminating news on TCV development. Keeping members informed and connected was vital leading up to the WHO and Gavi’s policy decisions on the TCV in 2017 and 2018.

## Social Media and Blog

As with many diseases, the challenge in typhoid advocacy has been less about accumulating evidence than it has been about communicating that evidence in a clear and impactful way. CaT and TyVAC publish the Take on Typhoid blog to communicate important issues such as drug resistance, integrated approaches to prevention and control, and the new TCV vaccine to a broad audience. Beyond translating data into more easily understood blog posts, CaT works to put a face on a statistic to bring attention to and inspire action on an issue. When advocates speak of typhoid, they want decision makers to not only remember the numbers and rates, but also know the names and faces they represent. To shine a light on the human face of typhoid, CaT launched the “Stories of Typhoid” blog series in May 2016. This popular series features some of the 12 million individuals, mostly children, who suffer from typhoid every year. It has profiled families touched by typhoid in endemic countries including India, Bangladesh, Kenya, Malawi, Uganda, Pakistan, and Nepal. These stories explore the linkages between socioeconomic status, environment, and disease, illustrating how typhoid is interconnected with poverty, education, access to information, and natural disasters. The series has inspired CaT members to submit their own stories, as well as some they have come across in the field. Readership of the Take on Typhoid blog spans the globe, with readers visiting the website most often from India, Pakistan, Nepal, Nigeria, the United States, and the United Kingdom. The diversity of readership illustrates the wide dissemination of messaging about typhoid that helps raise visibility of the disease.

Reaching out to a less technical audience is an important aspect to increase awareness and interest in preventing typhoid. Take on Typhoid is active on digital social media platforms to interact with a broader audience across sectors and rapidly share information and resources. When TCV prequalification was announced by the WHO, the Take on Typhoid social media plan quickly rallied people from all corners of the typhoid community around a single topic and hashtag, receiving >16 000 Twitter impressions. The participation of well-known thought leaders from the Bill & Melinda Gates Foundation and Gavi, the Vaccine Alliance lent credibility to the fight against typhoid, and signaled high-level support for TCV introduction through their social media support. Social media allows Take on Typhoid a far-reaching platform to stay connected and engaged, drive followers to resources on the website, and encourage participation and action across communities.

## CONCLUSIONS

Building a coalition around typhoid has rallied champions for typhoid worldwide to raise awareness about the disease on the global health and development stage. CaT’s advocacy for typhoid combined with the Take on Typhoid partnership is effectively amplifying voices and gathering the research community as a call to action for the urgent need to prevent and control typhoid. By providing a platform to strengthen partnerships, share knowledge, and advocate for typhoid prevention and control, the work of CaT members has been disseminated and data have reached decision makers both globally and in endemic countries. As a result, the political will and momentum are increasingly in place to implement prevention and control interventions, including the TCV, in the poor communities that have historically been left behind. With increased attention and coordinated action, typhoid can finally become a disease of the past everywhere.

## Notes

***Financial support.*** This publication is based on research funded in part by grants from the Bill & Melinda Gates Foundation (OPP1182032 and OPP1151153).

***Supplement sponsorship.*** This supplement is sponsored by the Center for Vaccine Development and Global Health (CVD) at the University of Maryland School of Medicine.

***Potential conflicts of interest.*** All authors: No reported conflicts. All authors have submitted the ICMJE Form for Disclosure of Potential Conflicts of Interest. Conflicts that the editors consider relevant to the content of the manuscript have been disclosed.
